# Continuous entanglement distribution over a transnational 248 km fiber link

**DOI:** 10.1038/s41467-022-33919-0

**Published:** 2022-10-17

**Authors:** Sebastian Philipp Neumann, Alexander Buchner, Lukas Bulla, Martin Bohmann, Rupert Ursin

**Affiliations:** 1 Institute for Quantum Optics and Quantum Information Vienna, Boltzmanngasse 3, 1090 Vienna, Austria; 2 Vienna Center for Quantum Science and Technology, Boltzmanngasse 5, 1090 Vienna, Austria

**Keywords:** Quantum optics, Fibre optics and optical communications, Quantum information, Single photons and quantum effects

## Abstract

Reliable long-distance distribution of entanglement is a key technique for many quantum applications, most notably quantum key distribution. Here, we present a continuously working, trusted-node free international link between Austria and Slovakia, directly distributing polarization-entangled photon pairs via 248 km of deployed telecommunication fiber. Despite 79 dB loss, we observe stable detected pair rates of 9 s^−1^ over 110 h. We mitigate multi-pair detections with strict temporal filtering, enabled by nonlocal compensation of chromatic dispersion and superconducting nanowire detectors. Fully automatized active polarization stabilization keeps the entangled state’s visibility at 86% for altogether 82 h. In a quantum cryptography context, this corresponds to an asymptotic secure key rate of 1.4 bits/s and 258 kbit of total key, considering finite-key effects. Our work paves the way for low-maintenance, ultra-stable quantum communication over long distances, independent of weather conditions and time of day, thus constituting an important step towards the quantum internet.

## Abstract (web summary)

Fibre-based entanglement distribution represents a key primitive for quantum applications such as QKD. Here, the authors demonstrate it across 248 km of deployed fiber, observing stable detected pair rates of 9 Hz for 110 h.

## Introduction

Entanglement is the basis of many quantum applications^1–8^. The technically most mature of them, quantum key distribution (QKD)^9–11^, harnesses quantum correlations of entangled photons to produce cryptographic keys of provably unbreakable security^12,13^. A key challenge in this context is the establishment of continuously working, reliable long-distance distributions of entanglement. However, connections via satellites^14,15^ do not allow for interruption-free operation, and deployed fiber implementations have so far been limited to <100 km by losses^16^, a few hours of duty time^17,18^, or use trusted nodes^19,20^.

Fiber-based QKD systems offer stable operation, independence from meteorological conditions, substantially reduced maintenance effort, straightforward mitigation of finite-key effects^21^, and the use of already deployed telecommunication infrastructure. These advantages can compensate for their higher losses^22^ compared to satellite connections. Therefore, while intercontinental quantum connections will most likely be operated using satellites, shorter distances of several hundred kilometers can be covered by fiber links^19,20,23^. Metropolitan fiber networks deploying entanglement-based QKD additionally have the advantage of allowing fully connect many users in a straightforward fashion^17,24^, potentially on fibers used for internet traffic, wavelength-multiplexed with the classical signal^25^. Nevertheless, losses in the fibers, imperfect preparation of entangled states, chromatic dispersion (CD), polarization mode dispersion, and timing precision in the detection of single photons hinder stable operation over long distances.

Up until today, the longest distance for entanglement distribution in deployed fiber was along a single 96 km fiber between Malta and Sicily^16^; additionally, the same submarine cable was used for a round-trip connection of altogether 192 km^26^. The longest uninterrupted operation of entanglement distribution, using active stabilization, has been demonstrated to work for 6 h along a deployed 10-km link^18^.

In this work, we combine state-of-the-art equipment and optimal exploitation of our polarization-entangled photons’ quantum properties to demonstrate continuously operated entanglement distribution along a distance of 248 km of deployed telecom fiber, connecting Bratislava in Slovakia and St. Pölten via Vienna in Austria. To the best of our knowledge, this is the longest distance for real-world fiber-based distribution of entanglement up until today. Additionally, we show ground-based entanglement distribution for QKD in a real-life two-channel configuration, while one channel crosses the international border between Austria and Slovakia without any intermediary trusted nodes. Despite unprecedented total loss of 79 dB, we achieve entangled pair rates of 9 s^−1^ and asymptotic secure key rates of 1.4 bits/s on average. We continuously operate the link for 110 h by actively stabilizing the polarization in a highly efficient, nonlocal way, achieving a duty cycle of 75% and a total key of 258 kbit considering finite-key effects.

## Results

### Sender and receiver infrastructure

**Fig. 1:**
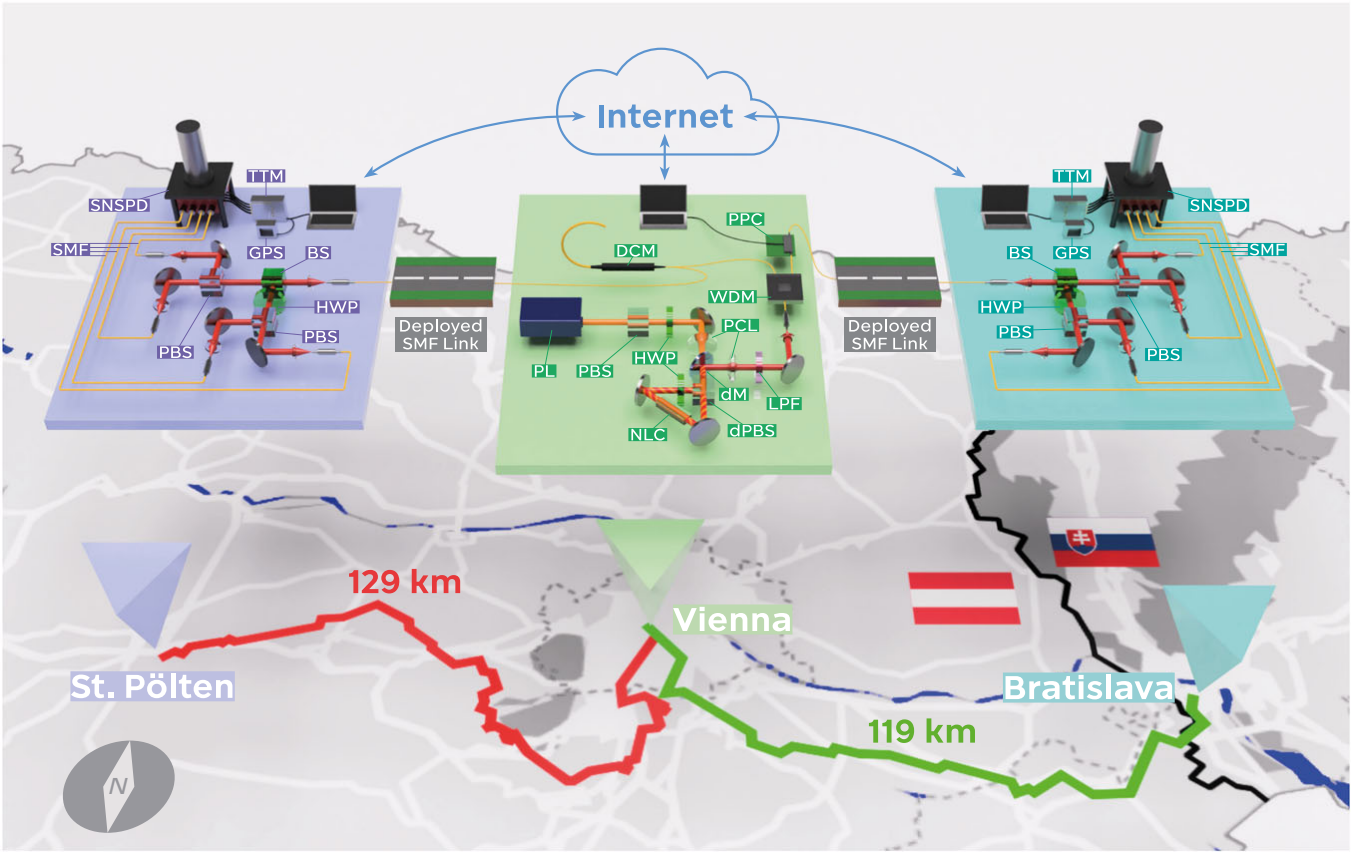
Sketch of the setup. The source of entangled photon pairs is situated in Vienna. We create polarization-entangled photon pairs at two distinct telecommunication wavelengths by pumping a non-linear crystal (NLC) in a Sagnac configuration with a 775 nm laser (PL) and collecting the down-converted photons with single-mode fibers (SMF) connected to a wavelength division (de-)multiplexer (WDM). The idler photon passes a dispersion compensation module (DCM), which nonlocally recovers the entangled state’s tight temporal correlations broadened by chromatic dispersion along the link. The idler is then directed along 129 km of fiber to a polarization measurement module (PMM) in St. Pölten in Lower Austria. The signal photon passes an automatized in-fiber piezo-based polarization controller (PPC), which nonlocally realigns the phase of the entangled state, should its quality decrease. Afterward, it travels to a PMM in Bratislava of the same design as the one in Austria. In these PMMs, the photons are randomly directed to orthogonal measurements in two mutually unbiased linear polarization bases. The photons impinge on superconducting nanowire single-photon detectors (SNSPD), and a GPS-clock-disciplined time-tagging module (TTM) records detection time, measurement basis, and outcome. Via classical internet connections, the two measurement stations’ detection events are compared and coincidences are calculated. If their quantum bit error rate increases, Vienna starts the polarization alignment. PBS polarizing beamsplitter, HWP half-wave plate, PL planoconvex lens, dPBS dichroic PBS, DM dichroic mirror, LPF longpass filter, BS 50:50 beamsplitter.

We implement a symmetric two-channel QKD system in a “source in the middle” configuration, following the BBM92 protocol^10^ for polarization entanglement (see Fig. 1 and the Methods section). The source of entangled photon pairs is situated in Vienna, utilizing continuous-wave-pumped spontaneous parametric down-conversion (SPDC) in a Sagnac configuration^27^ and wavelength division demultiplexing (WDM) to create entangled photons of 100 GHz spectral width around ~1550.12 nm with >99% fidelity to the Bell state

1 $$|{\phi }^{+}\rangle 	=1/\sqrt{2}(\,{|H \rangle }_{{{{{{\rm{SP}}}}}}}{|H \rangle }_{B}+{|V \rangle }_{{{{{{\rm{SP}}}}}}}{|V \rangle }_{B}) \\ 	=1/\sqrt{2}({|D \rangle }_{{{{{{\rm{SP}}}}}}}{|D \rangle }_{B}+{|A \rangle }_{{{{{{\rm{SP}}}}}}}{|A \rangle }_{B})$$

where H (V, D, A) refers to horizontal (vertical, diagonal, antidiagonal) polarization. The subscripts denote the receiver stations of the respective photon: St. Pölten in Lower Austria (SP) and the campus of the Slovakian Academy of Sciences in Bratislava (B). The connections are realized via deployed telecom fibers of 129 and 119 km length, respectively. The receivers measure each photon’s polarization state using a bulk polarization measurement module (PMM) in two mutually unbiased, randomly chosen linear polarization bases (H/V or, with equal probability, D/A). Superconducting nanowire single-photon detectors (SNSPD) connected to a time-tagging module (TTM) register each detection event. By comparing the detection times, SP and B identify the entangled photon pairs. The total transmission of each link was determined to be −40.2 dB (−38.4 dB) for the link to SP (B), including all losses and detection efficiencies. From this, it follows that an average of 8.9 pairs were detected per second between the receiver stations. We can only harness the quantum correlations of those pairwise (or “coincident”) events measured in the same polarization basis. The rate of these photon pairs is called “sifted” key rate and amounted to 4.4 s^−1^ on average in our case due to our passively implemented, balanced and random basis choice. Strict temporal filtering with a width of *t*_CC_ = 114 ps, also called the “coincidence window”, further reduces this value to 3.8 s^−1^. Of this rate, on average 0.34 coincidences originate from accidental counts, contributing 4.4 percent points to the quantum bit error rate (QBER), which is well below the 11.0% limit necessary to arrive at a non-zero key^28^.

### Nonlocal dispersion compensation

To reach such a low level of accidental coincidences over our high-loss link, sufficiently high temporal detection precision is necessary, allowing for strict temporal filtering^29^. The greatest effect detrimental to timing precision in our experiment is chromatic dispersion (CD) along the fibers. CD causes photons with finite spectral distribution to disperse in time, thus spreading the entangled photons’ temporal intensity correlation function *g*^(2)^. In our case, optical time-domain reflectometer measurements of the link yielded a CD of 16.8 (6.0) ps/nm/km at 1550 nm for the link to St. Pölten (Bratislava). Thus, our 100-GHz-bandwidth photons would suffer from a total CD of ~1.8 ns over the full fiber stretch, which is 50 times larger than the SNSPD and TTM jitters combined. To prohibit this, we deployed a single passive dispersion compensation module (DCM) with an equal and opposite CD of −1.8 ns acting on the photon traveling to St. Pölten only. Such nonlocal dispersion compensation^30–32^ harnesses the intrinsic quantum properties of entangled photon pairs to narrow their *g*^*(*2)^ distribution by acting on one photon of a pair only. The residual CD-induced temporal spread after compensation was masked by SNSPD jitter, TTM jitter, and GPS clock drift, which we identify as the remaining contributions to the overall timing uncertainty (see Methods section).

### Active polarization stabilization

**Fig. 2:**
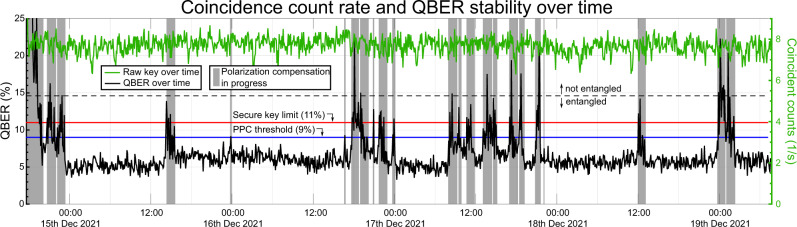
Quantum bit error rate (QBER) and coincident counts over time with a coincidence window of *t*_CC_ = 114 ps. When polarization drifts along the overland fiber link increase the QBER above 9%, the piezo-based polarization controller (PPC) starts an iterative alignment procedure to reduce the QBER below 7%. The limits were chosen such that alignment started well before the 11% limit of no key. Alignment takes between 8 min 10 sec and 1 h 22 min, and 57 min on average. We assume that the longer alignment phases are caused by polarization drift during alignment. The longest uninterrupted stable operation time amounted to 16 h 36 min. The coincidence counts stay at a constant value of ~7.7 s^−1^ over the full 110 h for *t*_CC_ = 114 ps.

Besides accidental coincidence counts, erroneous polarization measurements of (correctly identified) photon pairs contribute to the QBER. While such errors can be kept below 0.4% under laboratory conditions and for short time scales^33^, the nature of our experiment required active polarization stabilization of the altogether 248 km of deployed fibers, which were subject to polarization drift^34^. Without stabilization, this drift randomly changes the phase of the Bell state in Eq. (1), such that the quantum correlations between the photons at SP and B can no longer be observed with sufficient fidelity. To guarantee the long-term operation of our link despite this effect, we implemented an automatized algorithm for a piezo-based polarization controller (PPC) working on the fiber channel to Bratislava. It switched on whenever the QBER increased above 9% (see Fig. 2) and used the QBER value (calculated by B) directly as input, thus requiring no reference laser. Due to the nonlocal nature of the entangled quantum state, manipulation of just the photon in the B mode allows arriving at the correlations of Eq. (1).

The PPC iteratively scanned the voltage for each of the four fiber-squeezing piezoelectric crystals, thus optimizing the QBER via a hill-climb algorithm. Due to the low coincidence rates, it took the algorithm 2 min 32 sec on average to determine the QBER value with a precision of ±0.2%. Therefore, the mean length of the alignment procedure amounted to 57 min along the link, rather than sub-seconds in the laboratory, where coincidence rates were in the order of 10^4^ s^−1^. While the PPC is in operation, no key can be created, since both bit and basis information are sent via a classical internet connection. In our case, the PPC was active for altogether 27 h 57 min of 109 h 55 min, i.e., 25.4% of the time. This is equivalent to a duty cycle of 74.6% for the whole QKD scheme, with the longest uninterrupted operation time being 16 h 37 min. During this duty cycle, the QBER was kept at an average of 7.0%, where we attribute 2.6% to polarization measurement errors and 4.4% to accidental coincidences (see Methods section).

### Secure key rate analysis

**Fig. 3:**
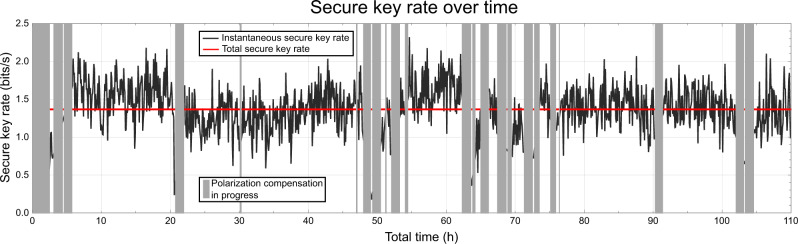
Secure key rate over time. As long as the quantum bit error rate (QBER) stays below 11%, a quantum secure key can be created in principle (see Fig. 2). Our polarization alignment procedure allows to keep the QBER in the key creation regime for altogether 82 out of 110 h of total link operation. The red line gives the average secret key rate (1.4 bits/s) calculated from all coincidences over these 82 h. Thus, the total asymptotic secure key amounts to 403 kbit, calculated with the overall, i.e., average, QBER. In black, we show the secure key rate based on data acquired over 300 sec time windows. Its fluctuations in the order of ±0.3 bits/s originate from Poissonian photon statistics and polarization drifts. For consideration of finite-key effects, we refer the reader to the Methods section.

To analyze the performance in a QKD setting, one has to consider the number of coincident clicks as well as their QBER. For the calculation of the final key rates, we follow the formula outlined in refs. 28, 35 (for details see the Methods section). Temporal filtering is of crucial importance for the final key size. Our optimal coincidence window *t*_CC_ amounted to 114 ps. It maximizes the total key accumulated over 110 h of link operation: 3.1 Mbit of the raw key with a QBER of 7.0% yield 403 kbit of quantum secure key, equivalent to a rate of 1.4 bits/s during the active time of 82 h (see Fig. 3). These values do not consider finite key effects. If taking them into account, the secure key over 110 h is reduced to 258 kbit, i.e., 64% of the asymptotic limit. Notably, this ratio approaches unity for longer integration times, and would already amount to 90% after three and a half months of continuous operation. To the best of our knowledge, our entanglement distribution link’s running time is only limited by the SNSPD maintenance cycle of ~10,000 h (i.e., close to 14 months).

## Discussion

We have shown an ultra-stable in-fiber polarization-based entanglement distribution scheme capable of creating quantum secure keys over a length of 248 km and a time span of 110 h, overcoming a total 79 dB of loss along two nearly symmetric fiber links. To this end, we deploy a high-brightness, high-fidelity source of entangled photon pairs at telecommunication wavelengths together with high-end SNSPD systems. We operate the link at the current limit of the state-of-the-art, which mainly originates from the precision of timing synchronization. We manage to lower it to 114 ps by non-locally compensating for the dispersion of our 100 GHz WDM channels and by the use of SNSPDs. We find coincidence rates in the order of 7.7 s^−1^ to be optimal to, on one hand, overcome loss and allow for live compensation of polarization drifts, and on the other hand to keep accidental coincidence rates sufficiently low.

Possible enhancements of our experiment could be realized by the use of several multiplexed wavelength channels, which could potentially increase the total key rates further^36^. Secondly, detection systems with lower detector jitter could allow for even stricter temporal filtering, which in turn enables higher pair production rates while keeping accidental coincidences low^29^. Thirdly, the PPC algorithm could be accelerated by automatically increasing the pump power during polarization alignment, which would yield better statistics for the QBER assessment. It might also allow for more stringent optimization, lowering the polarization-induced QBER below 1%. Fourthly, detailed trade-off calculations balancing the length of the polarization alignment with the quality of entanglement it establishes and the raw key block length it allows have to be carried out to determine optimal operation parameters. Fifthly, our entanglement distribution system could be integrated in wavelength-multiplexed quantum networks^17^, e.g., by implementing additional short fiber links to several users in Vienna. Sixthly, our analysis has mainly focused on QKD. The performance regarding other implementations, e.g., quantum computation or blind computing, still has to be evaluated and might have far-reaching implications.

Summarizing, our work paves the way for all kinds of continuously operated applications of quantum entanglement distributed over long fiber distances, most notably, but not limited to, quantum key distribution.

## Methods

### Source of entangled photon pairs

The strong attenuation along both fiber links requires a high-brightness source of polarization-entangled photon pairs in order to achieve significant coincidence rates between the receivers. To this end, we deploy a Sagnac-type source based on SPDC inside a 50 mm long bulk nonlinear ppLN crystal of type-0 phasematching pumped with a 775.06 nm continuous-wave Toptica laser. We choose a strong focusing parameter^37^ of *ξ* = 1.99 in order to arrive at pair production rates of 2.5 × 10^6 ^s^−1^/nm/mW (before all losses). The spatially degenerate entangled photon pairs are separated from the pump via a dichroic mirror and a longpass filter and coupled into one single-mode fiber. From the source’s 100 nm broad spectrum centered around 1550.12 nm, we select two 100 GHz WDM channels, using in-fiber add-drop multiplexers. The channel to SP (B) is centered at 1550.92 nm (1549.32 nm). Photons in one channel are entangled with their partner in the other channel due to energy conservation in the SPDC process^24^. The source was operated at 422 mW pump power, producing 6.4 × 10^8^ photon pairs per second. The source’s intrinsic QBER due to erroneous polarization measurements was determined to be less than 0.4% in a laboratory environment, and its collection efficiency for the 100 GHz WDM channels in use was ~26%.

### Single-photon polarization measurement

Two fiber links connect the source of entangled photons, located in the basement of the University of Vienna’s physics institute, to two measurement stations: A Türk Telekom repeater station in Getzersdorf, part of District St. Pölten in Lower Austria (SP), and the Research Center for Quantum Information on the campus of the Slovakian Academy of Sciences in Bratislava (B). The measurement apparatuses at SP and B are identical in construction (see Fig. 1). They each consist of a bulk PMM, a 4-channel SNSPD, and TTM. Local measurements in the laboratory without a long-distance link but including all losses in source, PMM, and SNSPD, have shown heralding efficiencies of ~20% on average, equivalent to −7.0 dB. All additional losses in our experiment can be attributed to the fiber links and compensation stages.

**Fig. 4:**
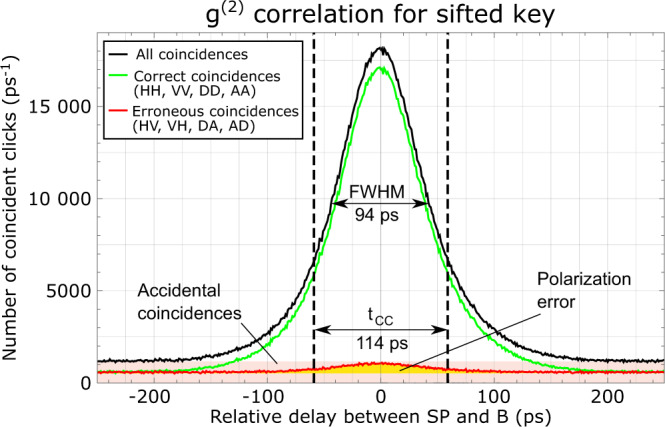
*g*^(2)^ intensity correlation of all coincidences used in key creation over the 82 h of stable QBER. The dotted line shows the width of the coincidence window, *t*_CC_ = 114 ps, yielding the highest total asymptotic key of 403 kbit. The ratio of the areas below the red and below the black curve within *t*_CC_ corresponds to the overall QBER of 7.0%. The yellow area depicts the number of erroneous measurements due to imperfect polarization alignment, amounting to ~2.6%. The remaining 4.4% of QBER can be attributed to accidental coincidences. We have subtracted the overall delay of ~44.8 μs from all relative delay values.

In the PMM, the photons are coupled out of the long-distance fiber and impinge on a 50:50 beamsplitter randomly directing them to two mutually unbiased linear polarization basis measurements. The first basis is realized by a PBS transmitting (reflecting) the horizontal (vertical) polarization mode, which is then coupled into one single-mode fiber each. The other basis, measuring the diagonal/antidiagonal basis, works alike except for an HWP set to 22.5° before the PBS, effectively rotating the polarization modes by 45°. The four PMM single-mode fibers are connected to the SNSPD, which detects photons with a probability of ~80% according to the manufacturer Single Quantum. All detection events are recorded using a TTM by Swabian Instruments with 1 ps resolution. The combined jitter of SNSPD and TTM on both sides amounts to ~38 ps full width at half maximum (FWHM). In order to identify detector clicks at SP and B originating from the same photon pair, each TTM is disciplined to a GPS clock. The relative drift of these clocks, on average 13 ps/s, limits the maximum integration time over which detection events at both receivers can be acquired and compared. As can be seen in Fig. 4, the polarization measurement error along the link (2.6%) is higher than in laboratory measurements (<0.4%) mainly due to our efforts to keep the PPC alignment time low, which did not allow us to set the entangled state perfectly for every alignment.

### Fiber link

Measurements with an optical time domain reflectometer (OTDR) of the fiber to St. Pölten (Bratislava) yielded a fiber length *L* of 129.0 km (119.2 km) and losses of −31.9 dB (−32.6 dB). Additional to loss, there are two dispersion effects detrimental to QKD imposed by long-distance fibers: CD and polarization mode dispersion (PMD).

CD is proportional to *L* and to the signal’s spectral width. It induces different travel times along the fiber for different parts of the light spectrum. This can effectively be seen as a decrease in temporal measurement precision, which smears out the correlation function between Alice and Bob, thus increasing the QBER and rendering live tracking impossible in our high-loss setting. In our experiment, we benefit from the fact that entanglement-based QKD allows for non-local dispersion compensation^30–32^. This means that the total CD effect of both fiber links can be reduced to zero by use of just one DCM. It introduces additional attenuation of ~−7 dB. OTDR measurements yielded a CD of 2073 ps/nm (617 ps/nm) to Alice (Bob). These values are so different because the fiber link to Bob partially conforms to the G.655 ITU standard, which allows less dispersion (6.0 ps/nm) than the more commonly used G.652 standard that was used for the link to Alice (16.8 ps/nm)^38^. Since ~24 km of fiber had not yet been connected to the link at the time the CD measurements were taken, we estimated the final overall CD to be 3.0 ns/nm, equivalent to 1.8 ns for our 100 GHz WDM spectra (with an FWHM of ~75 GHz ≈ 0.6 nm), and chose the DCM accordingly. The total FWHM of the correlation peaks amounts to 94 ps on average (see Fig. 4). We consider it a convolution of independent timing uncertainty effects: 38 ps originate from SNSPD and TTM jitter, as confirmed in the laboratory without link. The remaining 86 ps can be attributed either to the mean relative GPS clock drift accumulated over the post-processing integration time of 7 s^39^, or to residual uncompensated CD, or to both. In our realization, we had no means to differentiate between the two effects.

PMD causes different travel speeds for different polarization states inside the fiber. This is due to varying birefringence over the full stretch of the connection, which is in turn induced by random fiber imperfections. This effect scales with $\sqrt{L}$ since the accumulated imperfections can not only add up, but also cancel each other^40^. If PMD induces a temporal delay between two orthogonal polarization states which is larger than an unpolarized photon’s coherence time, it becomes polarized. In our case, this is equivalent to a polarization measurement and would therefore inhibit distribution of polarization entanglement^41^. OTDR measurements of the fiber to SP (B) have shown the PMD to be 0.63 ps (0.24 ps), which is substantially lower than our photons’ estimated coherence time of ≈10 ps. Accordingly, we could not observe PMD-induced loss of polarization fidelity along our fiber link.

### Data acquisition

Data was taken over the course of 109 h 55 min, starting on December 14th, 2021 at 17:38 and ending on December 19th, 7:05. The average count rates of all four detectors combined in SP (B) amounted to 62,500 s^−1^ (94,400 s^−1^), where 1200 s^−1^ each originate from detector-intrinsic dark counts. As a side remark, the dark counts are only responsible for ~3.2% of all accidental coincidences, or <0.3 QBER percent points—unavoidable single counts, i.e., photons which have lost their partner due to channel loss, are by far the more dominant QBER contribution.

Of all detector clicks, ~4.4 s^−1^ were coincident in the same measurement bases (“sifted key”) and can be used for key creation after error correction and privacy amplification. This number depends on the chosen coincidence window *t*_CC_: For live operation, we chose *t*_CC_ = 300 ps and an integration time of 9600 ms in order to register as many coincidences as possible. Note that these are the parameters used for polarization alignment and not those used for key creation since, for live operation, loss of tracking has to be prevented at all costs in order to ensure polarization stabilization. Such stable live operation of the system however relies on automatic temporal tracking of the coincidence peak, which is moving in time due to the GPS clocks’ relative temporal drift. If insufficient statistics, i.e., too few coincidences for the chosen integration time, cause the peak to be unrecognizable to the tracking algorithm, it can move out of the 1 ns monitoring window and be lost. This results in the failure of the protocol. On the other hand, if the integration time is too long, the clock drift can already start to smear out the coincidence peak, and no additional precision can be gained by integrating further. We chose the above parameters because they proved to work sufficiently well to not lose the tracking over the full measurement period, while still providing sufficient contrast for alignment. Thoroughly calculating the discussed trade-offs and optimizing the algorithm with regard to speed and effectiveness will be the subject of future studies. For key creation, which is done in post-processing, *t*_CC_ = 114 ps and an integration time of 7 sec—resulting in a sifted key rate of 3.8 s^−1^ and an average QBER of 7.0%—was shown to yield the largest key. For a detailed analysis of the choice of the optimal coincidence window, we refer the reader to the section “Asymptotic key rate calculation” and ref. 29.

### Active polarization stabilization

Polarization drifts along the deployed fibers due to stress, vibrations, and temperature changes constitute a challenge we overcome with the use of non-local polarization control. There have been approaches to automatize polarization drift compensation in both entanglement-based^42^ and prepare-and-send^43,44^ implementations, which, however, operated in regimes of substantially less loss and on shorter timescales. Our scheme was implemented in the B fiber, right after the source, via one PPC module. We align with respect to the QBER directly. No additional equipment such as a time- or wavelength-multiplexed reference laser has to be used in this scheme, which greatly reduces the engineering overhead of the experiment. Polarization drifts in both fiber links could be compensated with just one PPC due to the non-local nature of our entangled state. The algorithm in use optimized the visibility of the entangled state by iterative scanning of the voltages applied to the PPC’s four fiber-squeezing piezo-electric crystals. Since the coincidence rates used for live-tracking were only in the order of 5.3 s^−1^, the most time-consuming part of polarization optimization is accumulating enough statistics to determine the current visibility value with sufficient precision, which we chose to be ±0.2 QBER percent points, assuming Poissonian statistics. As can be seen in Fig. 2, the initial alignment takes much longer than the later corrections (more than 2 h 30 min). Subsequent alignments on the other hand can take as few as 8 min, and 57 min on average. We assume this is because the phase of the entangled state is completely random in the beginning. Polarization drifts, however, do not suddenly randomize the entangled state’s relative phase but only cause gradual changes which can be compensated faster.

We managed to compensate for only 25.4% of the total time, which is important since the coincidence data used for polarization alignment can naturally not be used for key creation. This also means that continuous monitoring of the QBER value, which we did for illustrative purposes in this publication, is not possible. Therefore, one can check the QBER values at certain points in time only, e.g., every hour. This would mean that the actual duty cycle of the system is reduced by another 3.1 percent points, if one assumes the determination of the QBER to take ~2:30 min. Additionally, if the QBER is found to be above the PPC limit, it might be beneficial to discard all data collected since the last QBER measurement in order to not dilute the overall raw key. This however depends on an estimate of how fast the QBER actually changes, and on how great the violation of the PPC limit was. Furthermore, the QBER limit for which the algorithm shall switch on also contributes to such optimization considerations—if the QBER limit is too low, the duty cycle can decrease drastically, if it is too high, then privacy amplification and error correction can consume substantial parts of the raw key. The 9% limit of our implementation was chosen via trial-and-error since we had no significant long-term polarization drift data available. Finite-key considerations ultimately also enter an optimization of polarization stabilization parameters, since both block size and QBER enter the final key length in a non-trivial way^45^. Our work can be the basis for detailed trade-off calculations, which are indispensable for commercially operated QKD systems and will be the subject of future studies.

**Fig. 5:**
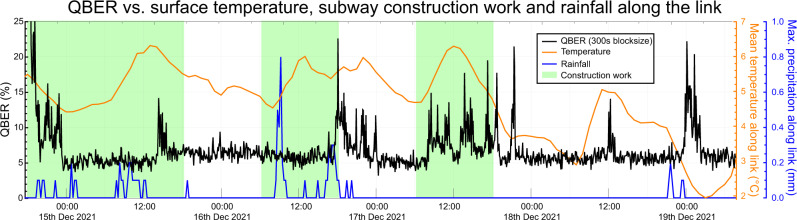
Depiction of QBER over time along with weather and construction site data. There was little variation in mean air temperature along the link and only short stretches of light rain. The construction site, whose operation times are depicted in green, was located close to the deployed fiber connecting the source with the overland fiber links. It involved subterranean drilling for the construction of a new subway line. We can find no convincing evidence for influences from weather and construction work on the polarization stability along the link. However, we cannot exclude destabilizing effects of possible additional construction sites along our 248 km link, since we had no data about them available. Also, harsher weather conditions might still result in polarization drift.

We also performed a preliminary investigation of possible environmental effects on the polarization stability (see Fig. 5). We compared QBER drifts with weather data by the Austrian Central Institution for Meteorology and Geodynamics (ZAMG) as well as with the working times at drilling sites for the future Viennese subway line U5, which we were supplied with by the Viennese public transport organization Wiener Linien. We find no convincing evidence for a correlation between any of the above effects and polarization drift. However, we want to emphasize the necessity of future studies regarding the impact of environmental factors on fiber-based QKD, especially for strong weather changes and over the course of the seasons.

### Asymptotic key rate calculation

The key rate depends on several experimental factors. Parameters like pump power, link loss, CD, fidelity and stability of the quantum state, coupling efficiency of the source, and the coincidence window have been carefully analyzed resp. chosen, following the methods outlined in ref. 29. For our calculation, we follow the key rate formula^35^

2 $${R}^{s}={R}^{sift}[1-2{H}_{2}(E)]$$

where *R*^s^ is the secure key rate in the limit of infinitely long raw key, *R*^sift^ is the sifted key rate, i.e., the rate of coincidences measured in compatible bases, and *H*_2_(x) is the binary Shannon entropy. Since both *R*^sift^ and *E* depend on *t*_CC_, one has to choose a coincidence window that neither excludes too many entangled pairs nor includes too many accidental counts (see Fig. 4).

The trade-off behind this calculation can be understood as follows: On one hand, if *t*_CC_ is too big, we unnecessarily include uncorrelated accidental counts in our valid coincidences, thus increasing the QBER and losing key to error correction and privacy amplification. On the other hand, if *t*_CC_ is chosen too small, too many valid coincidences get lost, and the size of our raw key decreases. Our numerical optimization of Eq. (2) leads to an optimal *t*_CC_ = 114 ps, which yields an asymptotic secure key with a length of 403,084 bits over 110 h of integration time.

In reality, the above trade-off further depends on the block size and the assumed error correction efficiency *f*. The latter we set to 1 in Eq. (2), since we assume an arbitrarily long key in our ultra-stable, actively compensated QKD scheme. If one however wants to divide the raw key into smaller blocks, *f* will increase^46^, which would in turn also lead to a different optimal value of *t*_CC_. Such smaller block sizes might be desirable due to the fact that in a real-life implementation, the QBER unavoidably fluctuates in time. In this case, it is beneficial to split a block of QBER E into n sub-blocks with *E*_1_,…,*E*_n_ where *E* = (∑^*n*^_*i* = 1_ *E*_i_)/n and calculate *R*^s^ as the sum of all *R*^s^_i_ (*E*_i_), since H_2_(x) is a concave function. On the other hand, smaller block sizes lead to less precise QBER estimates, effectively increasing the QBER, because one has to assume the worst *E* possible to guarantee a quantum secure key. Such trade-offs however are outside of the scope of this paper; we just present one asymptotic secure key length of 403 kbits for a single block containing the complete raw key, i.e., not exploiting the above considerations for shorter blocks.

### Finite key rate calculation

Block size also comes into play when taking into account security errors of parameter estimation, error correction, and privacy amplification during key creation. Insufficient raw key lengths are generally recognized to be a more pressing problem for satellite-based QKD with short measurement time intervals rather than for continuously operating fiber-based applications^21^. Nevertheless, any real-world application has to include finite-key calculations in their secure key estimates. For our calculations, we follow the formula outlined in ref. 21. and applied in ref. 47. while assuming a novel error correction method with ${{f}}\le 1.09$ as proposed in ref. 48.

The finite key length $l=\alpha m$ of our QKD implementation, which has acquired a raw key of length $m=$ 1,509,181 bits with an overall QBER *E* = 0.07, can be optimized by maximizing $\alpha \epsilon [{{{{\mathrm{0,\, 1}}}}}]$ while conforming to the inequalities

2 $$	{10}^{-\left(s+2\right)}+2\Bigg({{\exp }}\left[-\frac{2{m}^{2}\beta {\xi }^{2}}{m\left(1-\beta \right)+1}\right] \\ 	+ {{\exp }}{\left[-2\left(\frac{1}{m\left(E+\xi \right)+1}+\frac{1}{m-m\left(E+\xi \right)+1}\right)\left({m}^{2}{\left(1-\beta \right)}^{2}{\left(\upsilon -\xi \right)}^{2}-1\right)\right]\Bigg)}^{\frac{1}{2}} \\ 	+ \frac{1}{2}\sqrt{{2}^{-m\left(1-\beta \right)\left(1-{H}_{2}\left[E+\upsilon \right]-f{H}_{2}\left[E\right]\right)+{{{\log }}}_{2}\left[{10}^{s+2}\right]+\alpha m}}\le {10}^{-s}$$

and

3 $$0 \, < \,\xi < \,\nu\, < \frac{1}{2}-E$$

with $\beta$, $\xi$, and $\nu$ as free parameters. Here, $k=\beta m$ with $\beta \epsilon (0,\frac{1}{2}]$ amounts to the number of bits from the raw key used for parameter estimation, and *s*, which we set $s=9$ following ref. 47, is the security parameter giving a failure probability of ${{{{{{{\rm{\varepsilon }}}}}}}_{{QKD}}=10}^{-s}$ for the whole protocol. We numerically find the parameter values $\beta=$ 0.0964, ${{{{{\rm{\nu }}}}}}=$ 0.0127, and $\xi=$ 0.0116 to allow for the longest key, with $\alpha=$ 0.1711 and $l=$ 258,231 bits. This amounts to 64% of the key calculated in the asymptotic limit of infinite key length. If collecting data for 108 days in the configuration presented in this work, this value would reach 90%. When conservatively assuming a less efficient error correction protocol with ${{f}}\le 1.18$, $\alpha$ reduces to 0.1415 and thus $l=$ 213,493. Optimal duration of data collection will therefore depend on the final use case of the QKD link, and also on optimizations carried out with regard to trade-offs between polarization compensation fidelity goals and block length (see above).

## Data Availability

The time tag data files are available under restricted access due to their size of many terabytes, access can be obtained by contacting the corresponding authors.

## Code availability

The code used for the polarization alignment procedure is available from the corresponding authors upon request.
